# Einseitige Visusminderung bei beidseits randunscharfer Papille

**DOI:** 10.1007/s00347-020-01154-x

**Published:** 2020-07-06

**Authors:** Petra Dávidová, Ingo Schmack, Anna Slavík-Lenčová, Thomas Kohnen

**Affiliations:** Klinik für Augenheilkunde, Goethe-Universität Frankfurt am Main, Theodor-Stern-Kai 7, 60590 Frankfurt am Main, Deutschland

## Anamnese

Eine 47-jährige, adipöse Patientin westeuropäischer Herkunft stellte sich mit einer seit 1 Woche bestehenden, plötzlich aufgetretenen, schmerzlosen Visusminderung am linken Auge vor. Sie gab an, dass die Sehschärfe im Tagesverlauf allerdings schwanken würde. Zusätzlich hätte sie unter dem linken Auge eine diskrete, nicht druckschmerzhafte Verhärtung bemerkt. Das rechte Auge wäre dahingegen unauffällig. Allgemeinerkrankungen würden nicht bestehen trotz eines langjährigen Zigarettenkonsums (15 „pack years“).

## Klinischer Befund

**Figure 1:**
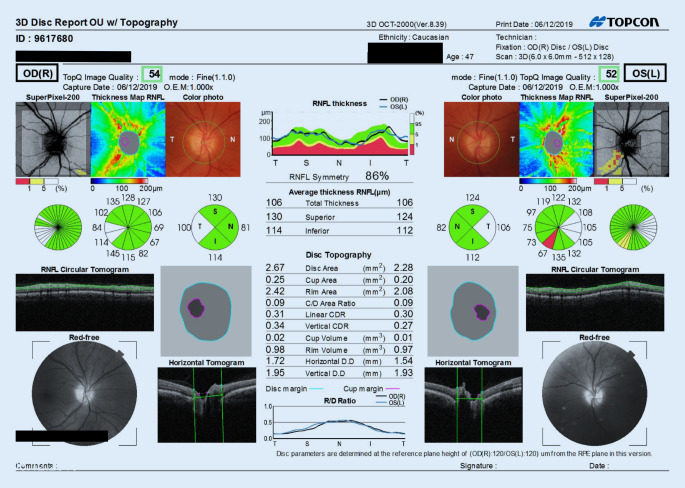

Die klinische Untersuchung ergab beidseits eine leichte Myopie sowie einen Astigmatismus (rechtes Auge: −2,00/−0,50/170°, linkes Auge: −1,00/−1,00/154°). Die Sehschärfe betrug bestkorrigiert auf dem rechten Auge 1,0 und auf dem linken Auge 0,63. Der Augendruck lag mit 18 (rechts) bzw. 21 mm Hg (links) jeweils im oberen Normbereich. Die vorderen Augenabschnitte waren beidseits unauffällig und regelrecht. Bei der Untersuchung des Augenhintergrundes zeigte sich jeweils eine leicht randunscharfe Papille mit nasal betonter Prominenz (Abb. 1). Die Sonographie (B-Scan) zum Ausschluss einer möglichen Drusenpapille sowie eine OCT-Untersuchung der Papillen waren mit Ausnahme einer diskreten Ausdünnung des Nervenfaserrandsaums links jedoch unauffällig (Abb. 1). Das linke Auge wies klinisch eine leichte Ptosis und einen Exophthalmus bei einer Seitendifferenz von 3,5 mm (Hertel Basis: 114 mm; rechts: 14 mm, links: 17,5 mm) auf. Orthoptisch zeigte sich eine Divergenzstellung von ca. 0,5 cm am linken Auge. Die Motilität war dennoch in allen diagnostischen Blickrichtungen frei und die Reagibilität der isokoren Pupillen regelrecht. Hinweise auf eine Farbsinnstörung inklusive Rotentsättigung oder einen Helligkeitsverlust lagen nicht vor. Das Blutbild war, abgesehen von einer geringen Erhöhung des CRP (1,28 mg/dl), ohne pathologischen Befund.

## Weiteres Procedere

**Figure 2:**
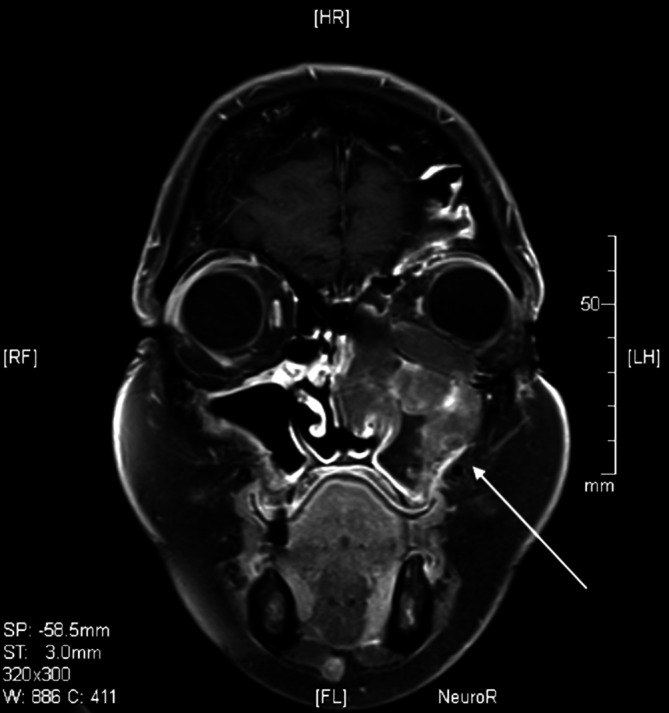

Zur weiteren diagnostischen Abklärung einschließlich Bildgebung wurde die Patientin stationär aufgenommen. In der Perimetrie fand sich am linken Auge eine Einschränkung der Gesichtsfeldaußengrenzen nach oben, die jedoch mit der vorhandenen Ptosis in Übereinstimmung zu bringen war. Skotome oder sonstige Auffälligkeiten zeigten sich nicht. Anhand der Magnetresonanztomographie des Schädels (Abb. 2) konnte jedoch eine kontrastmittelanreichernde, weichteilisointense Raumforderung links unter Einbeziehung des Sinus maxillaris, der vorderen Ethmoidalzellen, der Orbita und der Nasenhaupthöhle dargestellt werden, wobei eine Infiltration der Nasenmuscheln nicht sicher ausgeschlossen werden konnte. Zudem bestand eine Infiltration der Vorderwand des Sinus maxillaris und des paranasalen Weichteilgewebes. Zur weiteren Abklärung wurde durch die Kollegen der Hals-Nasen-Ohren-Klinik eine Biopsie aus der Nasenhaupthöhle und dem Sinus maxillaris links durchgeführt.

## Histologie

Man fand respiratorisches Schleimhautgewebe mit ortstypischen Drüsen, teilweise mit plattenepithelialer Metaplasie neben einem rasenartigen Proliferat von Zellen mit teils elongierten, teils gekanteten Kernen mit vergröberter Chromatinstruktur sowie schmalem Zytoplasmasaum. Dazwischen lagen Makrophagen und Nekrosen. Zudem zeigten sich eine Bcl2-Positivität und CD3-positive Blaseninfiltrate, die Tumorzellen waren deutlich positiv für CD56 und TIA1.

## Wie lautet Ihre Diagnose?

## Staging

Das anschließende Staging mittels CT-Hals, -Thorax und -Abdomen war unauffällig. Ebenso ergab die Knochenmarkpunktion zytologisch keinen Hinweis auf eine Generalisierung des Lymphoms. Das Tumorstadium wurde nach der Ann-Arbor-Klassifikation mit IE angegeben (lokalisierter Befall eines einzigen extralymphatischen Organs) [1]. Angesichts einer bekannten Kausalität zwischen EBV und dem Auftreten eines extranodalen NK/T-Zell-Lymphoms vom nasalen Typ und einer direkten Korrelation von zirkulierender EBV-DNS und dem Krankheitsverlauf wurde vorsorglich ergänzend der EBV-Status der Patientin laborchemisch bestimmt [2–5]. Das Ergebnis war in Bezug auf den Nachweis von IgG-Antikörpern (Titer: 851,3 AE/ml) und EBV-DNS im Plasma (CT-Wert 34,31) positiv. IgM-Antikörper wurden nicht nachgewiesen.

## Hintergrundinformationen

Insgesamt handelt es sich bei der vorgestellten Kasuistik um ein sehr seltenes Krankheitsbild, welches zur Gruppe der Non-Hodgkin-Lymphome gezählt wird [6]. Non-Hodgkin-Lymphome haben in Deutschland eine 10-Jahres-Prävalenz von 0,08 % [7]. Weltweit zeigt sich je nach geografischer Region ein mitunter differentes Verteilungsmuster. In Asien, Zentral- und Südamerika kommt das extranodale NK/T-Zell-Lymphom vom nasalen Typ deutlich häufiger vor als in Europa und Nordamerika. In den erstgenannten Regionen umfasst der Anteil dieser Lymphome unter allen Non-Hodgkin-Lymphomen bis zu 10 %. In Nordamerika und Europa hingegen sind es weniger als 1 %. Man geht in Europa sogar von einer Prävalenz von weniger als 1 bis 9 Fällen pro 1.000.000 Einwohnern aus [3–5, 8]. Hinsichtlich der Wahrscheinlichkeit ihres Auftretens besteht tendenziell zudem eine Prädisposition für das männliche Geschlecht. Am deutlichsten zeigt sich dies in Japan, wo bis zu 67 % der Erkrankten männlich sind [3–5, 9]. Das mittlere Erkrankungsalter liegt zwischen 44 und 54 Jahren [3–5].

Das extranodale NK/T-Zell-Lymphom vom nasalen Typ befällt v. a. die oberen Atemwege, insbesondere die Nasenhaupthöhle und breitet sich häufig in die Nasennebenhöhlen, Orbita, Nasopharynx, Oropharynx, Mundhöhle, Gaumen und in die Lymphknoten aus. Eine Infiltration des Knochenmarks ist selten. Die häufigsten klinischen Symptome umfassen Obstruktionen der Nase, Blutungen aus den oberen Atemwegen und eine B‑Symptomatik [3–5]. In fortgeschrittenen Stadien kann es zum Befall von Haut, Gastrointestinaltrakt, Leber, Lunge und Hoden kommen [3, 4].

Zur Behandlung werden verschiedene Therapieschemata herangezogen. Häufig werden bei lokalisierten Befunden kombinierte Radiochemotherapien nach z. B. DeVIC („Dexamethason, Etoposid, Ifosfamid, Carboplatin“) mit Bestrahlung von insgesamt 50 Gy angewandt. Bei fortgeschrittenen Stadien werden Schemata wie z. B. SMILE mit Verwendung von L‑Asparaginase angewandt „Dexamethason, Methotrexat, Ifosfamid, L‑Asparaginase, Etoposid“) [4, 5, 9].

Die Prognose des NK/T-Zell-Lymphoms ist insgesamt sehr variabel. Isolierte Formen des extranodalen NK/T-Zell-Lymphoms vom nasalen Typ haben insbesondere bei Verwendung einer kombinierten Radiochemotherapie nach oben genanntem Schema eine vielfach günstige Prognose mit 5‑Jahres-Überlebensraten von bis zu 72 % [4, 5, 9]. Zu den ungünstigen prognostischen Faktoren gehören, zusätzlich zu dem Nachweis von zirkulierender EBV-DNS und einem hohen Ki-67-Proliferationsindex (>40–65 %) Lymphozytenwerte <1000/µl, Hämoglobinwerte <11 g/dl, Thrombozytenwerte <150.000/µl und eine CRP-Erhöhung auf über 1 mg/dl. Zudem wirken sich ein fortgeschrittenes Krankheitsstadium (Stadium III/IV) sowie ein disseminierter Befall mit Beteiligung von Knochen oder Haut ebenso negativ auf den Krankheitsverlauf aus [3–5, 9]. Drei der genannten Kriterien erwiesen sich bei der beschriebenen Patientin als zutreffend. Wie bereits oben beschrieben, wurde zirkulierende EBV-DNS im Plasma mit einem CT-Wert von 34,31 und ein leicht erhöhter CRP-Wert von 1,28 mg/dl nachgewiesen. Hohe Titer der zirkulierenden EBV-DNS korrelieren allgemein mit einem fortgeschrittenen Tumorstadium, schlechtem Ansprechen auf die Therapie und geringeren Überlebensraten [3, 5]. Zudem lag eine Ki-67-Proliferationsrate, die knapp unter 90 % lag, vor. Weitere der oben beschriebenen Faktoren trafen nicht zu.

Durch die Einführung der oben genannten Therapieschemata, insbesondere SMILE, konnte die 5‑Jahres-Überlebensrate auch bei generalisierter Tumoraussaat insgesamt verbessert werden. Dennoch ist die 5‑Jahres-Überlebensrate bei NK/T-Zell-Lymphomen vom extranasalen Typ und disseminierten NK/T-Zell-Lymphomen vom nasalen Typ mit bis zu etwa 40 % weiterhin gering [3, 5, 9].

Die bei der Patientin vorhandene Ptosis des linken Oberlides führen wir auf eine isolierte Funktionsbeeinträchtigung des M. levator palpebrae superioris zurück. Sowohl der M. levator palpebrae superioris als auch der M. rectus superior werden beide durch den oberen Ast des N. oculomotorius (Ramus superior) innerviert. Der Ramus superior stellt dabei einen von 2 Hauptästen des N. oculomotorius dar, die sich nach dem Durchtritt des N. oculomotorius in die Orbita (Fissura orbitalis superior) von diesem abspalten. Angesichts der (1) MRT-Befunde, des (2) Fehlens von Motilitätseinschränkungen im Innervationsbereich des N. oculomotorius (trotz Bulbusverlagerung nach temporal-superior) sowie einer (3) intakten Pupillomotorik ist davon auszugehen, dass der Ptosis am linken Auge der Patientin ursächlich eine isolierte Kompression eines Endastes des Ramus superior zugrunde liegt.

## Therapie

Die Patientin erhielt eine kombinierte Radiochemotherapie, bestehend aus einer fraktionierten Photonenbestrahlung (Einzelreferenzdosis: 2 Gy; Gesamtreferenzdosis: 50 Gy) und einer intravenösen Gabe von Dexamethason, Etoposid, Ifosfamid und Carboplatin (DeVIC-Schema) über 3 Zyklen.

## Verlauf

Zwischen Diagnosestellung und Therapieeinleitung (ca. 3 Wochen) kam es am linken Auge zu einer fortscheitenden Visusverschlechterung von initial bestkorrigiert 0,63 auf 0,2. Zudem traten nun zusätzlich zur Bulbusfehlstellung nach temporal-oben Motilitätseinschränkungen mit einer Zunahme des Exophthalmus auf.

Bereits kurz nach Beginn der Therapie zeigte sich allerdings eine signifikant verbesserte Klinik mit einem Visusanstieg auf bestkorrigiert 1,0. Die Papillenrandunschärfe, die angesichts der diesbezüglich unauffälligen diagnostischen Befunde, rückblickend als physiologisch eingestuft wurde, verhielt sich dahingegen stabil. Ein kausaler Zusammenhang mit dem eigentlichen Tumorgeschehen konnte nicht hergestellt werden.

**Diagnose:** extranodales NK/T-Zell-Lymphom vom nasalen Typ

Die Patientin befindet sich gegenwärtig weiter in Therapie (3. Zyklus der Chemotherapie). In der Bildgebung zeigte sich bisher ein größenregredienter Befund, wobei die Patientin weiterhin eine Schwellung im Bereich der linken Infraorbitalregion und der Nasenhaupthöhle angibt.

## Fazit für die Praxis

- Eine ausführliche Anamneseerhebung mit Erfassung sämtlicher subjektiver Beschwerden ist oftmals bereits wegweisend für eine adäquate Befundeinordnung und zielführende, weiterführende Diagnostik.
- Der radiologischen Bildgebung kommt insbesondere bei diskreten, klinisch kaum fassbaren, jedoch verdächtigen Befunden ein hoher diagnostischer Stellenwert zu.
- Auch bei jungen Patienten sollte stets an eine onkologische Erkrankung gedacht werden.
- Die Beurteilung und Behandlung ophthalmologischer Krankheitsbilder bedarf oftmals einer eng abgestimmten interdisziplinären Zusammenarbeit.
